# Performance of *Aspergillus* Galactomannan Lateral Flow Assay on Bronchoalveolar Lavage Fluid for the Diagnosis of Invasive Pulmonary Aspergillosis

**DOI:** 10.3390/jof6040297

**Published:** 2020-11-18

**Authors:** Kathleen A. Linder, Carol A. Kauffman, Marisa H. Miceli

**Affiliations:** 1 Division of Infectious Diseases, Department of Internal Medicine, University of Michigan, Ann Arbor, MI 48109-5378, USA; linderk@med.umich.edu (K.A.L.); ckauff@med.umich.edu (C.A.K.); 2 Veterans Affairs Ann Arbor Healthcare System, Ann Arbor, MI 48105, USA

**Keywords:** invasive pulmonary aspergillosis, galactomannan, lateral flow assay, bronchoalveolar lavage fluid

## Abstract

**Background:** Several newly developed biomarker tests for invasive pulmonary aspergillosis (IPA) have been developed, including the IMMY *Aspergillus* galactomannan lateral flow assay (*Aspergillus* GM-LFA) evaluated in this study. **Methods:** Twenty patients with proven/probable IPA (EORTC/MSGERC criteria) were matched by age and underlying disease with 20 patients without IPA. Bronchoalveolar lavage fluid (BALF) was analyzed in duplicate using the *Aspergillus* GM-LFA. Results were read visually by two blinded observers, and the optical density index (ODI) was obtained digitally with a cube reader. **Results:** Using a cutoff of ≥0.5 ODI, the *Aspergillus* GM-LFA had a sensitivity of 40%, specificity of 80%, positive predictive value (PPV) of 67% and negative predictive value (NPV) of 57%. When the cutoff was increased to ≥1.0 ODI, sensitivity remained at 40%, specificity rose to 95%, PPV was 89%, and NPV was 61%. Excellent agreement was found when duplicate samples were read either visually (κ = 1) or with the cube reader (κ = 0.89). Correlation of results obtained by visual inspection and those obtained using the cube reader was excellent (κ = 0.82). **Conclusion:** The *Aspergillus* GM-LFA had poor sensitivity but excellent specificity for proven/probable IPA in BALF. The assay was easy to interpret, and there was high concordance between results obtained visually and with a cube reader.

## 1. Introduction

Diagnosis of invasive pulmonary aspergillosis (IPA) is challenging, and delays in diagnosis contribute to increased mortality. Noninvasive fungal biomarkers, such as the Platelia *Aspergillus* galactomannan (GM) enzyme immunoassay (EIA), which utilizes the single rat monoclonal antibody (MAb) EB-A2 to bind and detect GM, have proved useful in the diagnosis of IPA [1]. When performed on bronchoalveolar lavage fluid (BALF), the Platelia assay has shown increased sensitivity and specificity for IPA in patients who have a hematological malignancy, those who have received a hematopoietic cell or solid organ transplant, and patients with chronic pulmonary disease [2,3,4,5].

The IMMY *Aspergillus* galactomannan lateral flow assay (GM-LFA) is a newer non-culture-based diagnostic test that detects *Aspergillus* GM in serum and BALF. This rapid immunochromatographic test has the advantage of being able to provide point of care results in less than an hour. The *Aspergillus* GM-LFA utilizes two different MAbs, the ME-A5 MAb, which binds to a similar GM epitope as EB-A2 used in the Platelia GM EIA, and an undisclosed MAb. The second MAb, which detects other epitopes of GM, might enhance the sensitivity of the *Aspergillus* GM-LFA when compared with the Platelia EIA. However, detection of other epitopes also could lead to a decrease in specificity by allowing cross-reactivity with other fungi, such as *Scedosporium* species and *Fusarium* species, both of which can mimic IPA [6].

We sought to determine the test performance of the *Aspergillus* GM-LFA in BALF from a broad population of patients with risk factors for IPA, similar to how the test could be used in a general tertiary care hospital setting.

## 2. Materials and Methods

### 2.1. Patients and Setting

This retrospective, single-center study was performed at the University of Michigan Health System. It was approved by both the University of Michigan and the VA Ann Arbor Healthcare System Institutional Review Boards.

Residual BALF samples from bronchoscopy procedures performed for clinical care were prospectively collected from 9/2016 to 1/2020 and stored at −70 °C in the Infectious Diseases Laboratory at the VA Ann Arbor Healthcare System. The study population consisted of patients who had proven or probable IPA, including tracheobronchitis, as defined by the revised EORTC/MSGERC criteria [7]. These patients were matched 1:1 by age within 5 years and by host risk factors with control patients who did not have IPA. Whenever possible, the same host factors were used for matching, but in some cases, matching was accomplished by using other risk factors for IPA. Patients known to be colonized, but not infected with *Aspergillus* species or other fungi, were excluded from the study. Clinical data, including demographic information, comorbid conditions, immunosuppression status, radiological findings for invasive fungal infections, previously obtained results from the serum and BALF *Aspergillus* GM EIA (Platelia^TM^ *Aspergillus* EIA, Viracor-IBT Laboratories, Lee’s Summit, MO, USA), respiratory fungal cultures, and prophylactic antifungal agents were collected from the electronic medical record. All data were entered into a REDCap^®^ database.

### 2.2. Aspergillus Galactomannan Lateral Flow Assay

The *Aspergillus* GM-LFA (IMMY, Norman, OK, USA) was performed according to the manufacturer’s instructions. Briefly, 300 µL of BALF was mixed with 100 µL of pretreatment buffer, vortexed, heated at 120 °C for 6 min, and centrifuged at 14,000 g for 5 min. Then 80 µL of the pretreated BALF was mixed with 40 µL of *Aspergillus* GM-LFA running buffer, and a test strip was placed into the sample for 30 min before reading. Samples were run in duplicate in batches of 10 samples, and all readings were finished by 15 min after the end of the 30-min incubation time. Positive and negative controls provided with the test kit were run with each batch of samples.

An initial visual reading was performed by two independent observers, who were blinded as to the patient’s diagnosis. The test was read as negative if only the control line was present and as positive if both test sample and control lines were present. Positive tests were scored 1+, 2+, or 3+ based on the intensity of the test line. Optical density index (ODI) results were then obtained by placing those same strips into the *Aspergillus* GM-LFA cube reader, which was calibrated per the manufacturer’s instructions.

### 2.3. Statistical Analysis

Sensitivity, specificity, positive predictive values and negative predictive values for the diagnosis of proven/probable IPA were determined for BALF *Aspergillus* GM-LFA. Agreement between the duplicate test results read by visual inspection was evaluated using Cohen’s kappa coefficient (κ) with 95% CI, as was the agreement between the duplicate test results using the *Aspergillus* GM-LFA cube reader and the agreement between visual and digital readings for each sample. κ values below 0.6 represented poor agreement, values ≥0.6 and ≤0.8 represented good agreement, and values >0.8 were considered excellent agreement between readings. Statistical analyses were completed using SPSS software, version 26.0 (SPSS, Inc., Chicago, IL, USA).

## 3. Results

Of the 20 patients in the IPA cohort, 17 had probable and three had proven invasive pulmonary aspergillosis. The mean age of the 12 men and eight women was 55.1 ± 17.4 years. Host factors for IPA are noted in Table 1. The matched control cohort of 12 men and eight women, mean age 55.1 ± 17.1 years, had similar host factors for IPA (Table 1). Solid organ transplant recipients constituted almost half of each cohort. Only one patient with IPA had prior exposure to a mold-active antifungal agent. Radiological findings were consistent with those seen in IPA in 18 patients in the IPA cohort. Two additional patients in this cohort, both lung transplant recipients, had locally invasive *Aspergillus* tracheobronchitis at the anastomotic site. Fourteen patients in the IPA cohort had BALF cultures that yielded *Aspergillus* species (Table 2).

Using a cut-off value of ≥0.5 ODI, there were four false positive *Aspergillus* GM-LFA tests among the control cohort and 12 false negative results in the IPA cohort, giving a sensitivity of 40% and a specificity of 80%; the positive predictive value was 67% and the negative predictive value was 57% (Table 3). When ≥1.0 ODI was used as the cut-off, sensitivity remained at 40%, but specificity improved to 95%; the positive predictive value was 89%, and the negative predictive value was 61%.

There was excellent agreement (κ = 1) between the *Aspergillus* GM-LFA visual readings recorded as positive or negative and graded as 1+, 2+, or 3+ in the duplicate aliquots of each BALF sample. Similarly, the agreement between digital readouts for duplicate aliquots of each BALF sample was excellent, κ = 0.89 (95% CI 0.69–1.00). The agreement between the *Aspergillus* GM-LFA results obtained by visual inspection and those obtained using the cube digital reader was also excellent (κ = 0.82 (95% CI 0.58–1.00)) for both BALF aliquots.

In the IPA cohort, there was 100% concordance between the *Aspergillus* GM-LFA visual and digital results, irrespective of whether the cut-off value for positivity was ≥0.5 or ≥1.0. In the control cohort, there were several discrepancies between *Asp*ergillus GM-LFA visual and digital readings. In one case, when using a cut-off of ≥0.5 ODI, both aliquots of BALF samples were negative on visual inspection but positive by the cube digital reader. When the cut-off value was ≥1.0, these discrepancies were resolved, and all visual and digital readings were recorded as negative. In the only other discrepancy, both aliquots from one control BALF sample were read by both observers visually as 1+ but were recorded as barely positive (0.58 ODI) and negative (0.28 ODI) with the cube digital reader.

For the 10 BALF samples that were positive by both *Aspergillus* GM-LFA visual and digital readings, the semi-quantitative visual scoring method correlated well with the ODI readings. Specifically, both evaluators independently read samples that had ODI readings of 18.8–19.9 as 3+; those samples that had ODI readings of 2.9–6.7 ODI were reported as 2+; digital readings of 0.5–1.6 ODI were scored as 1+.

## 4. Discussion

Measurement of GM has become a valuable adjunct for the diagnosis of IPA. Most, but not all, investigators agree that measuring GM in BALF has enhanced sensitivity over measuring GM in serum, likely related to higher antigen levels present in the target organ than circulating in blood [2,3,4,5]. On the other hand, it has been noted that measuring GM in BALF can lead to more false positive test results depending on the population studied [8].

Several newer assays for the diagnosis of IPA have been developed, one of which is the *Aspergillus* GM-LFA developed by IMMY. One of the goals of this technology is to have a more rapid, reproducible assay that can be performed with a minimum of laboratory support. We noted that the test met those criteria. Interpretation of the lines on visual reading was simple and consistent among observers. Using the optical reader enhanced the precision of test interpretation by allowing quantitation of *Aspergillus* GM and appeared to be more accurate than visual reading when the amount of GM resulted in readings between 0.5 and 1.0 ODI. However, if one uses the revised EORTC/MSGERC criterion for a positive BALF GM being ≥1.0, the cube reader does not offer an advantage over the visual determinations.

Several studies have reported on the usefulness of the IMMY *Aspergillus* GM-LFA in serum or BALF [5,8,9,10,11,12,13]. When the assay was tested on stored sputum or BALF samples that had previously yielded *Aspergillus* species and that had been collected from a non-selected patient population, the sensitivity of the assay was 90%, but specificity was lower because of cross reactions with several other molds, including *Fusarium* and *Scedosporium* [5]. In one clinical study, the *Aspergillus* GM-LFA showed sensitivity and specificity as high as 89% and 100% when BALF from a small number of patients (*n* = 9) who had a hematological malignancy and proven or probable IPA were studied [9]. Further studies in a larger cohort of patients (*n* = 75) with a hematological malignancy and proven or probable IPA reported a sensitivity of 83% and a specificity of 87% when BALF was tested [10]. Others have reported lower sensitivity (69%) of *Aspergillus* GM-LFA on BALF from patients without underlying hematological disease [12]. Affirming that this assay appears to perform best in the population of patients who have a hematological malignancy, sensitivity and specificity as high as 97% and 98%, respectively, were reported when *Aspergillus* GM-LFA was evaluated using serum samples [11]. However, not all investigators have found the sensitivity of this assay when serum is used to be that high in patients who have a hematological malignancy; in a study that included 41 patients with probable or proven IPA, Mercier et al. reported that the sensitivity of the *Aspergillus* GM-LFA was only 49%, but specificity was 95% [13].

Our study is in agreement with most of those mentioned in regard to the high specificity of the *Aspergillus* GM-LFA when used in BALF [9,13]. We noted only one false positive test when the revised EORTC/MSG criterion of ODI ≥1.0 was used. In part, this high specificity could be because we excluded those patients who had been diagnosed with other invasive fungal infections and patients whose BALF cultures yielded *Aspergillus* species, but who met no other criteria for IPA.

Our study design stands out from the above studies, because we had a closely matched control group that we compared with the patients with proven and probable IPA. In our study cohort, only 25% of patients had an underlying hematological malignancy. We specifically wanted to assess the performance of the *Asp*ergillus GM-LFA in BALF of a broad population likely to have the test ordered in a general tertiary care medical center. Our study was designed to test the performance of the *Aspergillus* GM-LFA in BALF for the diagnosis of proven/probable IPA based on EORTC-MSGERC definitions and not to directly compare the *Aspergillus* GM-LFA with the Platelia GM EIA assay in BALF.

Limitations of our study include its single-centered, retrospective design and the relatively small number of patients with proven and probable IPA that was studied. Given the retrospective nature of the study, we were not able to control when bronchoscopy was performed to obtain the BALF samples in the course of the patient’s disease, and this could have influenced the sensitivity of detecting GM in BALF.

In conclusion, our study evaluated the performance of the IMMY *Aspergillus* GM-LFA on BALF in a cohort with well-characterized proven/probable IPA compared with a matched control cohort. We found that the BALF *Aspergillus* GM-LFA had poor sensitivity, but very high specificity for the diagnosis of IPA. The test system was simple to use, and results were highly reproducible.

## Figures and Tables

**Table 1:** Characteristics of patients with invasive pulmonary aspergillosis (IPA) and control patients who had bronchoalveolar lavage fluid samples tested with the *Aspergillus* galactomannan lateral flow assay.

	IPA Cases (*n* = 20) No. (%)	Controls (*n* = 20) No. (%)
Mean age (years ± SD)	55.1 ± 17.4	55.1 ± 17.1
Female	8 (40)	8 (40)
***Host factors for invasive Aspergillosis*** ^1^		
Solid organ transplant	9 (45)	9 (45)
Lung transplant	9	6
Liver transplant	0	2
Kidney transplant	0	1
High dose corticosteroids ^2^	4 (20)	4 (20)
Hematologic malignancy	5 (25)	4 (20)
Chronic pulmonary disease	2(10)	1 (5)
HIV <200 CD4/μL	1 (5)	1 (5)
Critical illness	2 (10)	2 (10)
***Additional features***		
Mold-active antifungal exposure ^3^	1 (5)	0

^1^ Several patients had more than one host factor; ^2^ dose equivalent to ≥0.3 mg/kg prednisone daily for ≥3 weeks at the time of bronchoscopy; ^3^ receipt of mold-active antifungal agent within 5 days prior to bronchoscopy.

**Table 2:** Demographics, underlying diseases, radiographic findings, and test results for 20 patients who underwent bronchoscopy and bronchoalveolar lavage and who were diagnosed with invasive pulmonary aspergillosis.

Age/Gender/ Comorbidity	Aspergillosis Category	GM BALF (ODI)	GM Serum (ODI)	*Asp* GM-LFA BALF (Visual Reading)		*Asp* GM-LFA BALF (Digital Reader)		BALF Culture	CT Chest Findings
				Test 1	Test 2	Test 1	Test 2		
70/F/Lung Tx	Proven at autopsy	NA	NA	+	+	1.41	1.63	*A. fumigatus*	Cavitary nodule, ggo, consolidation
42/M/ Cushing’s D., critical illness	Proven at autopsy	<0.5	<0.5	NEG	NEG	0.00	0.045	No growth	Pulmonary nodules, ggo
19/F/CPD	Proven at lobectomy	3.2	NA	+	+	2.13	2.63	*A. fumigatus*	Cavitary nodules, consolidation
67/M/Lung Tx	Probable	6.7	<0.5	NEG	NEG	0.43	0.34	*A. fumigatus*	Pulmonary nodules
22/M/AML	Probable	6.2	<0.5	NEG	NEG	0.06	0.01	No growth	Pulmonary nodules
42/M/Lung Tx	Probable	<0.5	NA	NEG	NEG	0.25	0.26	*A. fumigatus*	Pulmonary nodules
62/M/Lung Tx	Probable	<0.5	NA	NEG	NEG	0.00	0.06	*A. fumigatus*	Diffuse pulmonary infiltrates, ggo
23/F/Lung Tx	Probable	<0.5	<0.5	NEG	NEG	0.23	0.10	*A. fumigatus*	Pulmonary nodules
66/M/CMML	Probable	<0.5	1.75	NEG	NEG	0.00	0.09	No growth	Pulmonary nodules, ggo
79/F/CLL	Probable	NA	<0.5	NEG	NEG	0.13	0.23	*A. fumigatus*	Pulmonary nodules
58/F/ Steroids ^1^, critical illness	Probable	7.6	NA	++	+	3.16	2.88	NA	Pulmonary nodules
57/M/ HIV, CD4 < 200	Probable	7.4	<0.5	NEG	NEG	0.45	0.09	No growth	Consolidation, pulmonary nodules
49/F/Lung Tx	Probable ^2^	NA	NA	NEG	NEG	0.18	0.00	*A. fumigatus*	No pulmonary changes
68/M/MDS	Probable	2.43	<0.5	+	+	1.20	1.51	No growth	Cavitary nodules, ggo, consolidation
54/F/Steroids	Probable	8.14	<0.5	++	++	5.24	6.29	*A. fumigatus*	Cavitary nodule, ggo
63/M/Lung Tx ^3^	Probable	NA	<0.5	NEG	NEG	0.00	0.06	*A. fumigatus*	Pulmonary nodules
59/M/ Lung Tx, MDS	Probable	NA	NA	+++	+++	19.32	18.83	*A. fumigatus*	Pulmonary nodules
76/M/Steroids ^1^	Probable	NA	NA	++	++	5.97	6.73	*A. fumigatus*	Dense lesion with halo sign, consolidation
65/M/Lung Tx	Probable ^2^	NA	NA	+++	+++	19.9	19.43	*A. terreus*	Soft tissue density at anastomosis
60/F/CPD	Probable	NA	NA	NEG	NEG	0.34	0.37	*A. fumigatus*	Pulmonary nodules, ggo

^1^ Dose equivalent to ≥0.3 mg/kg prednisone daily for ≥3 weeks at the time of bronchoscopy; ^2^ tracheobronchial anastomotic infection; ^3^ patient on isavuconazole for prophylaxis within 5 days prior to bronchoscopy. +, ++, +++ refer to the strength of the positive test

**Table 3:** Evaluation of IMMY *Aspergillus* galactomannan lateral flow assay (*Asp* GM-LFA) on bronchoalveolar lavage fluid (BALF) from 20 patients with proven or probable invasive pulmonary aspergillosis and 20 control patients.

BALF *Asp* GM-LFA (ODI)	Test Performance							
	True (+)	False (+)	True (−)	False (−)	Sensitivity (%) (95%CI)	Specificity (%) (95%CI)	PPV (%) (95%CI)	NPV (%) (95%CI)
≥0.5	8	4	16	12	40 (19.1–63.9)	80 (56.3–94.3)	67 (41.7–84.8]	57 (46.71–66.9)
≥1.0	8	1	19	12	40 (19.1–63.9)	95 (75.1–99.9)	89 (52.4–98.3)	61 (52.2–to 69.6)

## Abbreviations

AML	acute myeloid leukemia
*Asp* GM-LFA	*Aspergillus* galactomannan lateral flow assay
BALF	bronchoalveolar lavage fluid
CLL	chronic lymphocytic leukemia
CMML	chronic myelomonocytic leukemia
CPD	chronic pulmonary disease
GM	galactomannan
ggo	ground glass opacities
HIV	human immunodeficiency virus
MDS	myelodysplastic syndrome
NA	not available
NEG	negative
ODI	optical density index
Tx	transplant

## References

[1] Maertens J., Miceli M.H. (2015). Role of non-culture-based tests, with an emphasis on galactomannan testing for the diagnosis of invasive aspergillosis. Semin. Respir. Crit. Care Med..

[2] Zhou W., Li H., Zhang Y., Huang M., He Q., Li P., Zhang F., Shi Y., Su X. (2017). Diagnostic value of galactomannan antigen test in serum and bronchoalveolar lavage fluid samples from patients with nonneutropenic invasive pulmonary aspergillosis. J. Clin. Microbiol..

[3] Boch T., Buchheidt D., Spiess B., Miethke T., Hofmann W.-K., Reinwald M. (2015). Direct comparison of galactomannan performance in concurrent serum and bronchoalveolar lavage samples in immunocompromised patients at risk for invasive pulmonary aspergillosis. Mycoses.

[4] Clancy C.J., Jaber R.A., Leather H.L., Wingard J.R., Staley B., Wheat L.J., Cline C.L., Rand K.H., Schain D., Baz M. (2007). Bronchoalveolar lavage galactomannan in diagnosis of invasive pulmonary aspergillosis among solid-organ transplant recipients. J. Clin. Microbiol..

[5] Maertens J., Maertens V., Theunissen K., Meersseman W., Meersseman P., Meers S., Verbeken E., Verhoef G., Van Eldere J., Lagrou K. (2009). Bronchoalveolar lavage fluid galactomannan for the diagnosis of invasive pulmonary aspergillosis in patients with hematologic diseases. Clin. Infect. Dis..

[6] Lass-Flörl C., Cascio G.L., Nucci M., Dos Santos M.C., Colombo A.L., Vossen M., Willinger B. (2019). Respiratory specimens and the diagnostic accuracy of *Aspergillus* lateral flow assays (LFA-IMMY ™): Real-life data from a multicentre study. Clin. Microbiol. Infect..

[7] Donnelly J.P., Chen S.C., A Kauffman C., Steinbach W.J., Baddley J.W., E Verweij P., Clancy C.J., Wingard J.R., Lockhart S.R., Groll A.H. (2020). Revision and update of the consensus sefinitions of invasive fungal disease From the European Organization for Research and Treatment of Cancer and the Mycoses Study Group Education and Research Consortium. Clin. Infect. Dis..

[8] Farmakiotis D., Le A., Weiss Z., Ismail N., Kubiak D.W., Koo S. (2018). False positive bronchoalveolar lavage galactomannan: Effect of host and cut-off value. Mycoses.

[9] Jenks J.D., Mehta S.R., Taplitz R., Law N., Reed S.L., Hoenigl M. (2019). Bronchoalveolar lavage Aspergillus Galactomannan lateral flow assay versus *Aspergillus*-specific lateral flow device test for diagnosis of invasive pulmonary aspergillosis in patients with hematological malignancies. J. Infect..

[10] Mercier T., Dunbar A., De Kort E., Schauwvlieghe A., Reynders M., Guldentops E., A Blijlevens N.M., Vonk A.G., Rijnders B., E Verweij P. (2020). Lateral flow assays for diagnosing invasive pulmonary aspergillosis in adult hematology patients: A comparative multicenter study. Med. Mycol..

[11] White P.L., Price J.S., Posso R., Cutlan-Vaughan M., Vale L., Backx M. (2020). Evaluation of the performance of the IMMY sona *Aspergillus* galactomannan lateral flow assay when testing serum to aid in diagnosis of invasive aspergillosis. J. Clin. Microbiol..

[12] Jenks J.D., Mehta S.R., Taplitz R., Aslam S., Reed S.L., Hoenigl M. (2019). Point-of-care diagnosis of invasive aspergillosis in non-neutropenic patients: *Aspergillus* galactomannan lateral flow ssay versus *Aspergillus* -specific lateral flow device test in bronchoalveolar lavage. Mycoses.

[13] Mercier T., Gukdentops E., Lagrou K., Maertens J. (2020). Prospective evaluation of the turbidometric beta-D-glucan assay and 2 lateral flow assays on serum in invasive aspergillosis. Clin. Infect. Dis..

